# A novel HRM assay for the simultaneous detection and differentiation of eight poxviruses of medical and veterinary importance

**DOI:** 10.1038/srep42892

**Published:** 2017-02-20

**Authors:** Esayas Gelaye, Lukas Mach, Jolanta Kolodziejek, Reingard Grabherr, Angelika Loitsch, Jenna E. Achenbach, Norbert Nowotny, Adama Diallo, Charles Euloge Lamien

**Affiliations:** 1 Animal Production and Health Laboratory, Joint FAO/IAEA Division of Nuclear Techniques in Food and Agriculture, Department of Nuclear Sciences and Applications, International Atomic Energy Agency, Wagramer Strasse 5, P.O. Box 100, A-1400 Vienna, Austria; 2 Institute of Applied Genetics and Cell Biology, University of Natural Resources and Life Sciences, Muthgasse 18, A-1190 Vienna, Austria; 3 Research and Development Department, National Veterinary Institute, P.O. Box 19, Debre Zeit, Ethiopia; 4 Viral Zoonoses, Emerging and Vector-Borne Infections Group, Institute of Virology, University of Veterinary Medicine, Veterinaerplatz 1, A-1210 Vienna, Austria; 5 Institute of Applied Microbiology, University of Natural Resources and Life Sciences, Muthgasse 11, A-1190 Vienna, Austria; 6 Institute for Veterinary Disease Control, Austrian Agency for Health and Food Safety, Robert Koch-Gasse 17, A-2340 Mödling, Austria; 7 Department of Basic Medical Sciences, College of Medicine, Mohammed Bin Rashid University of Medicine and Health Sciences, Dubai Healthcare City, P.O. Box 505055, Dubai, United Arab Emirates

## Abstract

Poxviruses belonging to the Orthopoxvirus, Capripoxvirus and Parapoxvirus genera share common host species and create a challenge for diagnosis. Here, we developed a novel multiplex PCR method for the simultaneous detection and differentiation of eight poxviruses, belonging to three genera: cowpox virus (CPXV) and camelpox virus (CMLV) [genus *Orthopoxvirus*]; goatpox virus (GTPV), sheeppox virus (SPPV) and lumpy skin disease virus (LSDV) [genus *Capripoxvirus*]; orf virus (ORFV), pseudocowpox virus (PCPV) and bovine papular stomatitis virus (BPSV) [genus *Parapoxvirus*]. The assay is based on high-resolution melting curve analysis (HRMCA) of PCR amplicons produced using genus specific primer pairs and dsDNA binding dye. Differences in fragment size and GC content were used as discriminating power. The assay generated three well separated melting regions for each genus and provided additional intra-genus genotyping allowing the differentiation of the eight poxviruses based on amplicon melting temperature. Out of 271 poxviral DNA samples tested: seven CPXV, 25 CMLV, 42 GTPV, 20 SPPV, 120 LSDV, 33 ORFV, 20 PCPV and two BPSV were detected; two samples presented co-infection with CMLV and PCPV. The assay provides a rapid, sensitive, specific and cost-effective method for the detection of pox diseases in a broad range of animal species and humans.

Poxviruses are responsible for medically and economically important diseases of human beings and animals worldwide. They are large, complex DNA viruses of the *Poxviridae* family, which is further subdivided into subfamilies *Chordopoxvirinae*, with poxviruses infecting vertebrates, and *Entomopoxvirinae*, infecting invertebrates^1^,^2^. The *Chordopoxvirinae* subfamily has been subdivided into eight genera: Orthopoxvirus, Parapoxvirus, Capripoxvirus, Suipoxvirus, Molluscipoxvirus, Avipoxvirus, Leporipoxvirus and Yatapoxvirus.

Most poxviruses are classified based on the name of the host species from which the sample was originally collected even though, some poxviruses have a broader host range^2^. Chordopoxviruses infections are characterised by the induction of localized or generalized “pox” lesions on the skin of the affected animals^2^.

The genome of poxviruses consists of a single molecule of linear double-stranded DNA varying in size from 130–375 kbp. It encodes a large number of proteins, allowing the virus to replicate with a considerable degree of autonomy from its host^2^,^3^. Poxviral genomes vary significantly in their GC content, although, the evolutionary factors for this divergence are unknown. For instance, capripoxviruses and orthopoxviruses have low GC content (approximately 25% and 33%, respectively) while parapoxviruses exhibit a high total GC content of 64%^4^,^5^.

The current study focuses on eight viruses belonging to three genera of the *Chordopoxvirinae* subfamily: cowpox virus (CPXV) and camelpox virus (CMLV) [genus Orthopoxvirus]; goatpox virus (GTPV), sheeppox virus (SPPV) and lumpy skin disease virus (LSDV) [genus Capripoxvirus]; orf virus (ORFV), pseudocowpox virus (PCPV) and bovine papular stomatitis virus (BPSV) [genus Parapoxvirus]. Capripoxviruses, parapoxviruses and orthopoxviruses are all able to infect ruminants, with the orthopoxviruses and parapoxviruses having in addition the potential to infect both camels and humans.

Within the genus Orthopoxvirus, CMLV causes severe infections of economic importance only in camels, with rare incursions in humans^6^. Camel pox is present in Africa, the Middle East, and southwestern Asia^7^. Cowpox virus has a much wider host range, in addition to cattle. Indeed, CPXV appears to be classified inappropriately since rodents are the reservoir of this virus, and cats occasionally transmit it to humans^8^. Cowpox is enzootic in Europe and Russia^9^,^10^.

Capripoxvirus infections of ruminants cause significant morbidity and mortality in Africa, the Middle East and Asia. Recently, their incursions into Eastern Europe have been reported^7^,^11^,^12^. While SPPV and GTPV infect mainly small domestic and wild ruminants, LSDV infection seems to be restricted to cattle and large wild ruminants.

Parapoxviruses infect a wide range of hosts worldwide and are of both economical and medical importance, infecting mainly persons working in close contact with animals. Within the parapoxvirus genus, ORFV infects small ruminants, camels and humans^13^,^14^,^15^, while BPSV and PCPV infect large ruminants^16^, with the latter being able to also infect camels^17^ and humans^18^,^19^.

As some of these poxviruses have similar geographical locations and can infect the same animal species (i.e., ORFV, SPPV and GTPV cause generalized or localized cutaneous lesions in both sheep and goats), this can cause problems for clinical diagnosis of these diseases. Additionally, poxvirus infections can be clinically confused with other cutaneous diseases, and other poxviruses are emerging or re-emerging in various parts of the world^5^. This creates the need for updated discriminatory diagnostics for quick and accurate identification of the pathogen(s).

While capripoxviruses and orthopoxviruses can be differentiated from parapoxviruses by electron microscope based on the differences in their shape, such a differentiation cannot be done between capripoxviruses and orthopoxviruses, and not at all species level. Serologically, it is almost impossible to discriminate between species within the same genus. However, the development of molecular diagnostics offers the opportunity to target genomic signatures of the pathogens and discriminate between relatively closely related pathogens even within the same species^20^. Additionally, molecular diagnostics methods offer the possibility for multiplex assay design targeting several pathogens in a single reaction.

Several approaches were used to differentiate poxviruses based on classical or real-time PCR^5^,^21^,^22^. For real-time PCR design, probe-based approaches could be avoided by using high-resolution melting curve analysis (HRMCA)^23^. This approach, which enables genotyping and mutation scanning for clinical applications, is a fast, cost-effective, sensitive, specific and high-throughput single-nucleotide polymorphism (SNP) detection and genotyping method^23^,^24^,^25^,^26^,^27^,^28^,^29^,^30^.

The purpose of the present study was to develop and evaluate a novel multiplex assay based on the HRM principle as a rapid, low-cost and technically simple method for the simultaneous detection and differentiation of eight poxviruses of veterinary and medical importance worldwide.

## Results

### Assay design

**Figure 1:**
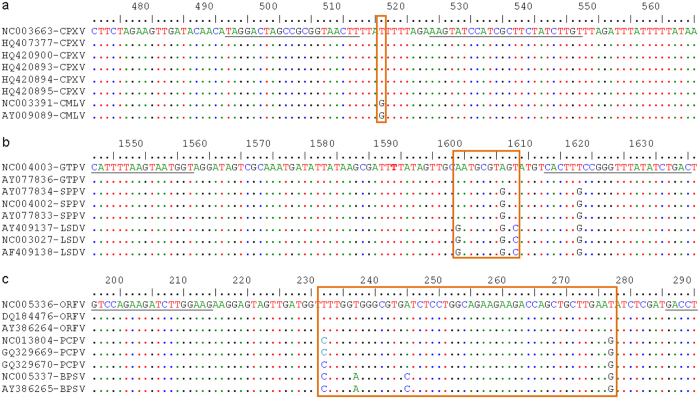
Nucleotide alignment of the targeted gene sequences of poxviruses. Nucleotide mismatches in each genus are highlighted with orange colored box. (**a**) Orthopoxviruses show T:G mismatch between CPXV and CMLV. (**b**) AAT:AGT:GGC nucleotide variations exist for GTPV, SPPV and LSDV of capripoxviruses. (**c**) Parapoxviruses show TTAT:CTAG:CACG nucleotide mismatches for ORFV, PCPV and BPSV, respectively. The forward and reverse primer sequences are underlined flanking the nucleotide variations. Identical nucleotides are shown as dots in reference to the first sequence.

The current assay was designed to simultaneously detect and differentiate members of three different genera of poxviruses (Orthopoxvirus, Capripoxvirus and Parapoxvirus) and to provide additional genotyping of the viruses within each of the three genera. Thus, a different target was selected for each of the three genera in the way to amplify small fragments of the virus genome in a region where nucleotide genetic differences exist within the genus. The target region was identified by aligning the nucleotide sequences of the full genome of orthopoxviruses, capripoxviruses and parapoxviruses available in GenBank (Fig. 1). Primers flanking the species-specific signature region were selected in the conserved area of the target for each genus. An important aspect of this strategy was to select primers to amplify fragments of different lengths for each genus. The smallest fragments were targeted for orthopoxviruses and capripoxviruses, with lowest GC content, in a way that the multiplex detection using double stranded DNA intercalating dyes could generate amplicons with significant differences in melting temperature for each genus. Thus, the orthopoxvirus fragment with 56 nucleotides had a total GC content of 37%, followed by the capripoxvirus fragment (100 bp, 38% GC content) and the parapoxvirus fragment (112 bp, 51% GC content). Additionally, the nucleotide differences between the members of each of the genera allowed the generation of PCR amplicons with a different melting temperature for each species within the genus.

Using this strategy, we were able to produce amplicons with three different melting regions corresponding to the three virus genera, and different Tm values among the targeted genotypes within each genus. Thus, we produced eight unambiguous melting profiles for the eight targeted poxviruses.

### PCR optimization

The initial evaluation and optimization was performed in a singleplex reaction for each genus using a plasmid harbouring the target fragments for each considered virus. In the initial evaluation, the best primer pair was selected for each of the three genera and optimized. This involved the testing of various primer pairs to avoid an overlapping or similar Tm of the tested viruses. In a second stage, the three primer pairs, one for each genus, were pooled to produce a multiplex assay.

When using these six primers in a single reaction, we have detected primer-dimers that appeared with Tm values similar to that of PCR amplicons. Troubleshooting of this issue revealed that the concentration of primers, and the annealing temperature and time are the critical parameters to consider. Thus, we have tested different conditions before achieving the optimized detection and differentiation protocol presented in the materials and methods section. The temperature and time for heating and cooling of the PCR products were also optimized in order to achieve more accurate results.

### Melting temperature and HRMCA

Employing the fluorescent melting curve analysis using CFX96Touch^TM^Real-Time PCR Detection System (Bio-Rad), we categorized the eight poxviruses into three regions based on their Tm of PCR products: orthopoxviruses, capripoxviruses and parapoxviruses which showed low (72.00–73.40 °C), medium (75.60–77.40 °C) and high (79.80–81.60 °C) Tm values, respectively (Table 1).

Furthermore, within the genera, each species revealed a unique Tm that helped to differentiate the tested virus at the genus and species level in a single PCR reaction.

**Figure 2:**
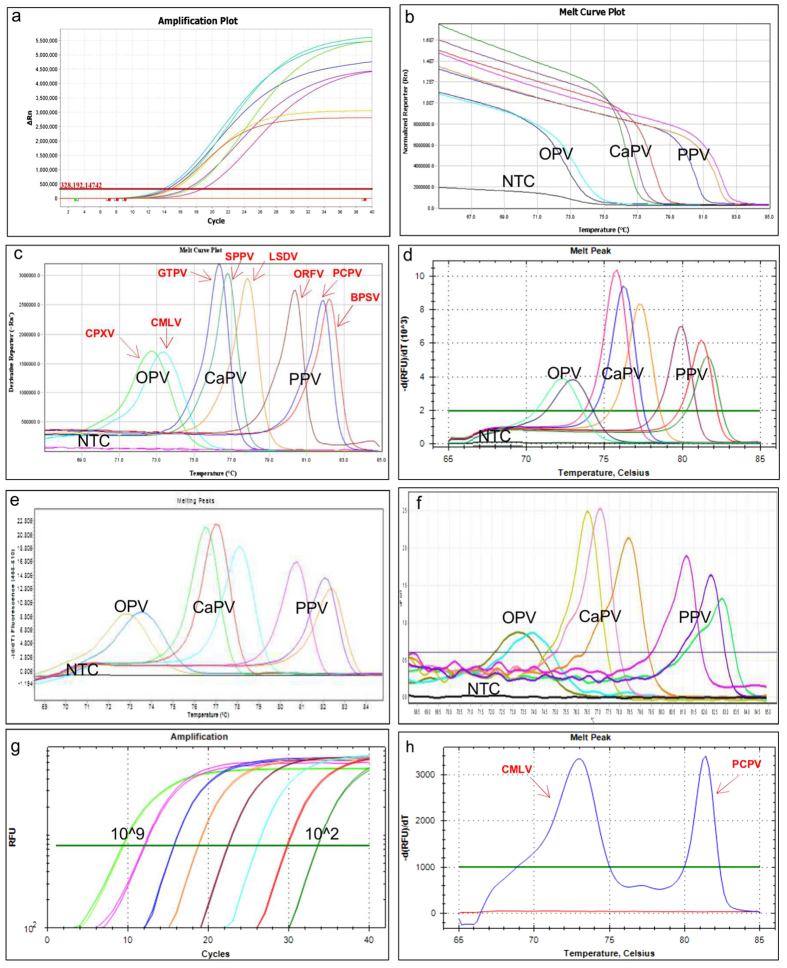
Melting curve analysis of the targeted eight poxviruses using different PCR platforms. Each genotype displayed a unique melting peak. (**a**). amplification plot; (**b**). melt curve plot; (**c**). melting peaks using the QuantStudio 6, Life Technologies); (**d**). melting peaks using the CFX96, Bio-Rad; (**e**). melting peaks using LC480II, Roche; (**f**). melting peaks using the Rotor Gene Q, Qiagen); (**g**). Linearity test (10^9^ to 10^2^ virus copies); and (**h**). Co-infection with CMLV and camel PCPV (blue colour two melting peaks, 72.80 °C and 81.20 °C for CMLV and camel PCPV respectively).

At the species level, using a serial dilution of the constructed plasmid (10^2^ to 10^6^ copies/reaction), the following melting temperatures were recorded: CPXV (72.35 ± 0.09 °C), CMLV (73.00 ± 0.00 °C), GTPV (75.88 ± 0.10 °C), SPPV (76.35 ± 0.09 °C), LSDV (77.35 ± 0.09 °C), ORFV (80.35 ± 0.09 °C), PCPV (81.32 ± 0.10 °C) and BPSV (81.60 ± 0.00 °C), confirming that the differentiation of these eight poxviruses is possible in one PCR reaction without the need of using fluorescently labelled probes (Fig. 2). Although, the classical melting curve analysis was sufficient to achieve the simultaneous detection and differentiation of the eight poxviruses, we used the HRM software to analyze the melting of the PCR amplicons. The plots of the difference relative fluorescence unit versus temperature of the PCR products were analyzed individually for each genus by selecting the corresponding active melt region located between the pre-and post-melt regions of the melting curve.

**Figure 3:**
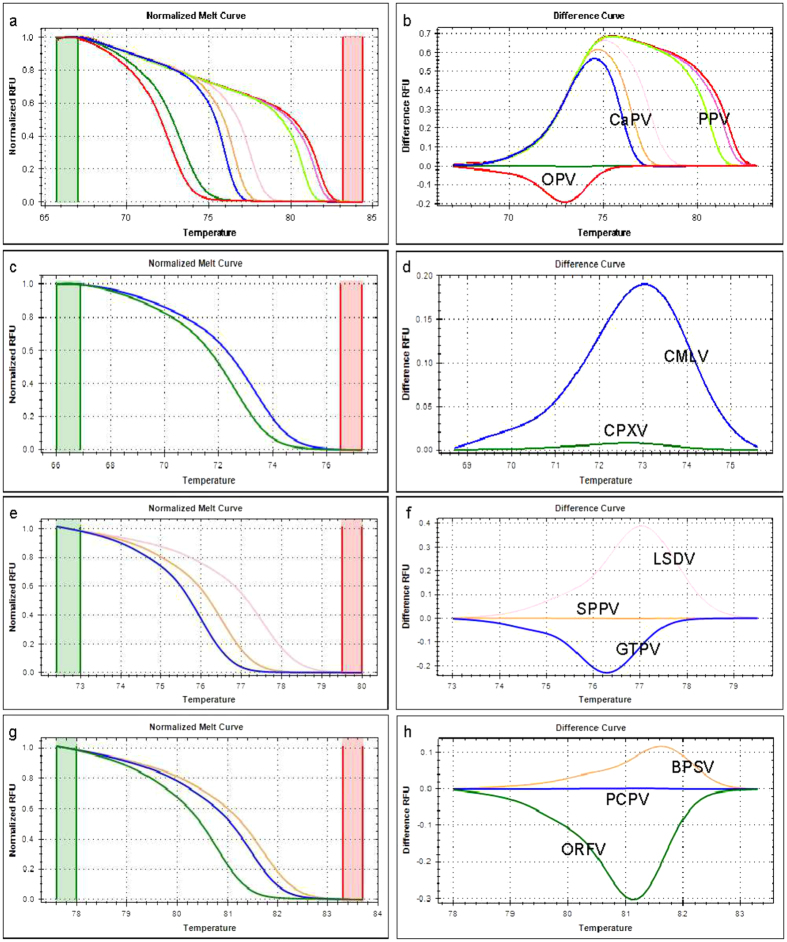
Normalized HRM plots of the PCR products of eight poxviruses. Three primer pairs were used for the amplification. Each virus genotype clustered separately within the genus. The normalized melt curve and difference curve plots are presented separately with different line colour for each genotype within the genus: for the eight poxviruses (**a**,**b**), orthopoxviruses (**c**,**d**), capripoxviruses (**e**,**f**), and parapoxviruses (**g**,**h**), respectively. Green and red columns in the normalized melt curve plot represent pre- and post-melt normalization regions.

The HRM analysis results were in agreement with the classical melting curve analysis, although, a clearer view of the separation between the species was observed by clustering and assigning different colours for each genotype (Fig. 3).

### Limit of detection of the assay

The lower limit of detection (LOD) of the current method was determined using the probit analysis. Five replicates of different dilutions, 100 copies to 10 copies, of plasmids carrying the appropriate fragment for each viral species, were amplified on three separate occasions, and the proportion of positive results was determined for each concentration. For each of these eight poxviruses, an accurate species identification was possible at relatively low concentrations; the limits of detection, expressed as number of copies per reaction, at a 95% confidence were 13.05 [9.95–16.22], 15.07 [11.88–18.67], 15.00[12.29–17.72], 10.33[7.01–13.66], 10.33[7.01–13.66], 13.99 [11.06–16.99], 15.07 [11.88–18.67], and 31.42 [25.58–38.31] for CPXV, CMLV, GTPV, SPPV, LSDV, ORFV, PCPV and BPSV respectively.

### Cross-platform compatibility test

To evaluate the cross-platform compatibility of the method, we performed the assay under the optimized PCR conditions described in the materials and methods section using the relevant plasmids and also virus DNA extracted from clinical material and cell culture supernatants in four different real-time PCR instruments. We succeeded to accurately detect and assign each of the eight poxviruses to the correct species. However, there was a constant but small shift in the Tm values of the amplicons from one instrument to another as indicated in Table 1. Furthermore, after performing the amplification steps using the classical PCR machine (Bio-Rad C1000) and transferring the plates containing the amplified product to the CFX96™ real-time PCR system (Bio-Rad) only for melting curve analysis, we have successfully assigned each virus to the correct species.

### Specificity, discriminating power of the assay

**Figure 4:**
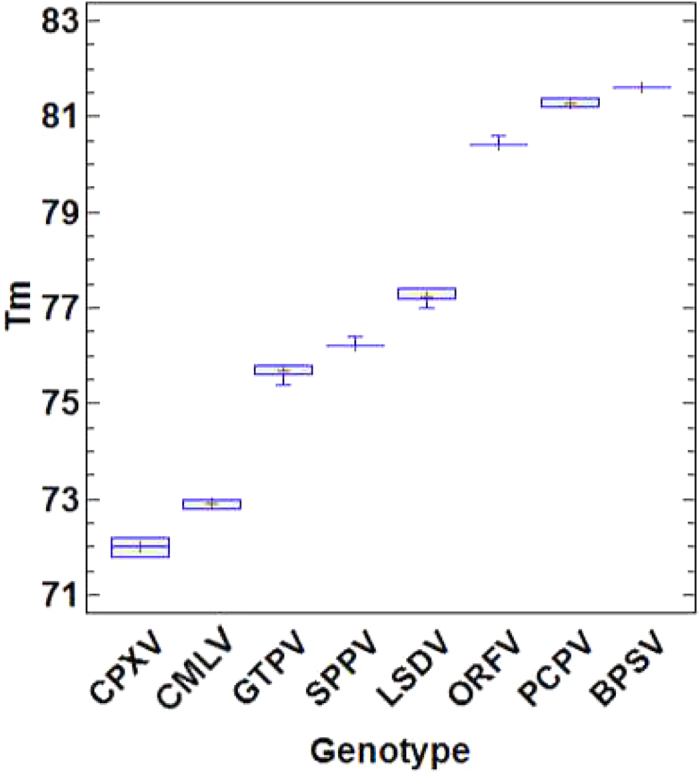
Box and whisker plot showing the melting temperatures (Tm) of the targeted eight poxviruses. Note that only two sample were available for BPSV.

To study the discriminating power of the assay, DNA extracted from clinical specimens (n = 219), collected from pox disease suspected animals (215) and humans (4) as well as infected cell culture supernatants (n = 52) were tested (Supplementary Table S1). Out of 271 DNA samples tested (known = 128 and blind = 143) using the current assay (Supplementary Table S1) the following poxviruses were detected: CPXV (n = 7), CMLV (n = 25), GTPV (n = 42), SPPV (n = 20), LSDV (n = 120), ORFV (n = 33), PCPV (n = 20) and BPSV (n = 2); additionally, two samples contained both CMLV and PCPV. The One way ANOVA test showed that the average Tm were significantly different (P = 0.0000) between each of the eight poxvirus genotypes. The overall Tm ranges with each of the genotypes while using field samples and cell cultures isolates are illustrated in (Fig. 4). Additionally, to demonstrate the ability of the assay to detect poxviruses directly in swab samples, nasal swab (n = 41), ocular swab (n = 1) and lymph node aspirate (n = 14) samples, where included in the 120 samples collected from clinically diseased cattle during lumpy skin disease suspected outbreaks in Ethiopia. The results confirmed that the sampled cattle were infected with LSDV.

To further evaluate the specificity of the method, non-poxviral DNA samples from CCPP, and cDNA from PPRV and FMDV (Supplementary Table S2) were tested. No DNA amplification was recorded. Another unique feature of this assay was its ability to detect co-infection in clinical samples. This was proven by simultaneously detecting CMLV and camel PCPV (causative agent of camel contagious ecthyma, CCE) in two skin lesion samples collected from camels in Ethiopia (Fig. 2). This co-infection was confirmed by sequencing the major envelope protein (B2L) and hemagglutinin (HA) genes for camel PCPV and CMLV, respectively.

### Practical application of the assay for pox disease investigations in Ethiopia

To demonstrate the usefulness of the assay, we used the assay to systematically screen old and new outbreak samples collected from diseased ruminants and camels presenting pox-like lesions.

In 2008, a pox disease outbreak occurred in sheep at the Adami-tulu agriculture research center (ATARC), four months after vaccination of the animals with a capripox vaccine. At the time, skin scraping samples were submitted by the researchers to the NVI, Ethiopia, with the suspicions of capripoxvirus infections. The initial testing of these samples using capripoxvirus specific primers revealed all samples to be negative for capripoxvirus. Investigations did not go further as NVI did not have other means to look for the causative agent. These samples were retrospectively screened in 2013 using the newly developed assay described in this manuscript. Interestingly, all samples collected from the ATARC outbreak were found positive for ORFV. This result was then confirmed using ORFV specific primers^31^ and sequencing^32^.

In 2011, 2012 and 2014 there were outbreaks of pox disease in camels in the Afar, Somali and Borena regions of Ethiopia, respectively, and camelpox was suspected. Nodular skin lesion samples collected from camels showing pox-like lesions were submitted to NVI for virus confirmation. All samples collected from the outbreaks including those which were tested negative using a CMLV-specific PCR^33^ were retrospectively screened using the current assay. The results showed PCPV DNA was present in ten of the tested samples (Afar six samples, Somali three samples and Borena one sample).

From the twenty-seven CMLV-infection suspected samples originating from the Borena and Somali regions of Ethiopia, 2011–2014, tested with the method described here, two samples revealed co-infections with CMLV and PCPV as shown in Fig. 2. Thus, the practical application of the current detection and differentiation method was evaluated with these three practical examples collected from different animal species representing the three genera of poxviruses.

## Discussion

This paper describes the development of a real-time PCR method for the simultaneous detection and accurate differentiation of eight different poxviruses. The method is targeted towards important poxviruses of ruminants and camels including those of public health importance, namely, CPXV, CMLV, GTPV, SPPV, LSDV, ORFV, PCPV, and BPSV.

First, a unique target offering the possibility for intra-genus differentiation was selected to design three assays, to simultaneously detect and differentiate CPXV from CMLV, GTPV from LSDV and SPPV, and ORFV from BPSV and PCPV.

In general, parapoxviruses have the highest GC content among the poxviruses, followed by orthopoxviruses and capripoxviruses^4^,^5^. However, in our design, we targeted fragments of similar GC content for orthopoxviruses and capripoxviruses, with orthopoxviruses having the shortest amplicon lengths, so that they displayed the lowest melting temperatures. The above mentioned singleplex assays were assessed, merged, and optimized to constitute the final multiplex assay.

This resulted in a powerful assay which unequivocally detected and assigned each of the analysed poxvirus suspected DNA samples into the appropriate genus, and furthermore to the corresponding species. The results were in complete agreement with the previously developed species-specific detection methods for CaPVs^34^,^35^, ORFV^31^ and CMLV^33^, or were validated by sequencing the relevant PCR products for CPXV, PCPV and BPSV.

The assay was highly specific, with no inter-species cross-reactivity among the different poxviruses and no reactivity to other ruminant viral and bacterial pathogens tested in this study. Additionally, the assay displayed good sensitivity making it suitable as a screening tool during pox disease outbreak investigations.

As this assay does not require the use of a probe or labelled primers, is easy to set-up and interpret with a straight-forward analysis of the melting data; it can easily be implemented in laboratories with moderate resources. Another advantage is that the method is very fast, since the complete PCR protocol needs only 85 minutes or less depending on the PCR platform used.

By allowing the simultaneous detection and discrimination between eight poxviruses in a single PCR reaction, this assay stands as an ideal front-line method for pox-like disease screening, saving time and reducing cost, while providing an accurate identification of the responsible pathogen without the need for partial sequencing of the pathogen’s genome.

Interestingly, the current method provided similar discriminating performances, across platforms, based only on the Tm values of the melting curves, without the need to use the HRM data analysis software. Indeed, the only variation observed was a constant shift in Tm values across the platforms for each of the eight viral species, possibly due to the variability in the fluorescence data collection mode and data analysis software. Nevertheless, the use of HRM software to analyze the melting of the PCR amplicons provides better intra-genus discrimination. Due to its cross platform compatibility the current method presents the potential for easy implementation in a number of veterinary and public health diagnostic laboratories.

The main weakness of the current method is that only two BPSV samples were available for analytical validation. However, from the publicly available sequences of BPSV (NC005337, AY386265), it is expected that more BPSV would be detectable by the assay. We also noticed that the sensitivity of the assay for capripoxviruses was slightly lower than the real-time PCR-based genotyping assay for capripoxviruses using dual hybridization probes^34^.

Poxvirus infections of ruminants, camels and humans are widely distributed worldwide with very complex epidemiological pictures in some countries where several poxviruses of different genera and species are present.

Within the Orthopoxvirus, Parapoxvirus and Capripoxvirus genera, a single species can infect several different hosts with the characteristic occurrence of nodules on the skin of the affected host. This creates a huge challenge for the diagnosis and management of pox diseases at both public health and veterinary levels, especially when several of these poxviruses are present in the same geographical location.

For instance, this study revealed the misdiagnosis of ORFV infection in sheep in Ethiopia, where the disease was confounded with SPPV and GTPV infections. A similar scenario can easily happen in other African, Asian or Middle Eastern countries where SPPV, GTPV and ORFV, capable of affecting both sheep and goats, are all present^11^,^12^,^36^,^37^,^38^,^39^. The same is applicable for European countries, such as Greece and Bulgaria, where SPPV has re-emerged and ORFV is already endemic.

We also demonstrated that two outbreaks of PCPV infections in camels were misdiagnosed in Ethiopia, due to the confusion with CMLV infections, probably because of a better awareness of camelpox disease in the region, and the lack of an appropriate rapid differential diagnostic method.

Although, no case of misdiagnosis of skin infection in cattle was found in this study, it is expected that the presented assay can also help to elucidate poxviruses infections in cattle worldwide. For example, the differentiation of LSDV from PCPV, CPXV and BPSV is becoming an urgent matter in the Middle East and Europe due to the recent entry of LSDV on these two continents^12^.

Finally, this tool will allow the rapid identification of zoonotic infections of humans by poxviruses, such as CPXV, ORF, CMLV and PCPV. The more rapid the diagnosis, the quicker an appropriate treatment can be applied.

Frequently, misdiagnoses of poxvirus infections are due to the absence of appropriate diagnostic techniques and in case specific tests are available, they mostly exist only in a single-plex format, requiring multiple testing until the causative pathogen is identified. In laboratories with limited resources, only assays targeting those pathogens of more significant economical and public health importance are made available, therefore, many of the poxvirus infections may remain undetected, creating a bias in the estimation of disease prevalence in a specific area.

To address those challenges, several multiplex assays were recently developed for poxviruses^21^,^40^,^41^. However, compared the assay presented in this paper, the above-mentioned assays exhibit the limitation of being agarose gel based or the need of using probes, which results in additional time and costs. Moreover, the assays focused on a limited number of poxvirus species: (i)^21^ focused on variola, monkeypox, cowpox, and vaccinia viruses (all orthopoxviruses); (ii)^41^ targeted SPPV, GTPV and ORFV; while (iii)^40^ differentiated ORFV, PCPV and BPSV.

The field applications presented in this study show the usefulness of this assay in resolving differential disease diagnosis issues, by clearly demonstrating that two suspected camelpox outbreaks were actually PCPV infections of camels. Additionally, its potential as a dual or co- infection diagnosis tool was also established. These two advantages will be very useful for the discovery of unexpected hosts, when used for outbreak investigations and to discover potential reservoir hosts in wildlife.

Here we describe a novel multiplex real-time PCR approach for poxvirus detection and typing exploiting both GC content and fragment lengths combined with high resolution melting principle. The assay is reproducible, sensitive and specific and was able to simultaneously detect and classify eight poxviruses of public health and veterinary importance. Since this assay is cost-effective and easy to perform, we recommend it as a rapid screening and confirmatory tool for poxvirus infections during outbreak investigations, disease epidemiology studies, poxviral vaccine identity evaluations and wildlife screening. Since most of the poxviruses of this panel are classified as notifiable diseases by WHO and OIE, it is expected that this assay significantly contributes to pox disease management at both, the public health and veterinary level.

## Materials and Methods

### Viruses and nucleic acid extractions

The poxviruses used in this study are listed in Supplementary Table S1. Pathological tissues suspensions (10% w/v) were prepared in sterile phosphate buffer saline (PBS). Each of the swab samples was re-suspended in 0.5 ml PBS. DNA was extracted from 200 μL of pathological tissues suspensions, swab suspension or infected cell culture supernatant using DNeasy^®^ Blood and Tissue kit (Qiagen, Germany) following the manufacturer’s instruction. The DNA was eluted in 100 μL of elution buffer and kept at −20 °C until further analysis.

### Targeted genes and Primers design

The full genomes of eight representatives of each of the three poxvirus genera were downloaded from GenBank (accession numbers are indicated in Fig. 1). This included: six CPXVs and two CMLVs viruses [Orthopoxviruses]; two GTPVs, three SPPVs and three LSDVs [Capripoxviruses]; three ORFVs, three PCPVs and two BPSVs [Parapoxviruses]. The members of each of the genera were aligned separately to scan for potential intra-genus genotyping targets using MAFFT (http://mafft.cbrc.jp/alignment/server). The targets were selected to achieve the three following objectives: (1) produce PCR amplicons with similar size, but enough nucleotide differences to provide intra-genus genotyping within each of the three genera; (2) produce PCR amplicons of different sizes and overall GC content to provide enough separation between the melting regions of the three genera under consideration; (3) to provide after multiplexing a clear separation of the eight poxviruses included in the panel. The following genes were selected for the development of the current assay: the ssDNA-binding phosphoprotein gene (I3L) of orthopoxviruses, the RNA polymerase subunit gene (RPO147) of capripoxviruses, and the late transcription factor gene (VLTF3) of parapoxviruses. Primers were designed on the conserved regions flanking the nucleotide variations (Fig. 1) using Allele ID version 6 software (Premier Biosoft International, Palo Alto, CA, USA) and synthesized by Eurofins Genomics, Austria. The specificity of each primer sequence was checked by using the Basic Local Alignment Search Tool (blastn; http://blast.ncbi.nlm.nih.gov./Blast.cgi). Accordingly, six non-modified or non-labelled primers were produced (Table 2). The total G + C content of the predicted PCR amplicons (Table 2) was calculated using BioEdit software package version 7.1.3.0^42^.

### Positive control production

Capripoxviruses (GTPV-Denizli, SPPV-Denizli and LSDV-Ismalia), orthopoxviruses (CMLV- Hadow/01/2012 and CPXV-72/93), and parapoxviruses (ORFV- DZ C-1, PCPV-2200/12 and BPSV- Stamm M1) were used for the production of positive control plasmids (Table 3). Thus, for plasmid production the targeted full gene was amplified by PCR using the primers indicated in Table 4, for each representative of these eight poxviruses except for capripoxviruses, where only a partial sequence of the RPO147 gene containing the PCR fragment was amplified. The purified PCR products were cloned into pGEM^®^-T Vector system (Promega) and the plasmids were sequenced commercially by LGC genomics, to confirm the presence of the correct target. The concentration of each plasmid was determined fluorometrically using Quant-iT PicoGreen dsDNA Assay Kit (Invitrogen) and a NanoDrop 3300 Fluorospectrometer (Thermo Scientific) and converted into copy numbers as previously described^34^.

### PCR and Melting Curve Acquisition

The PCR was set up in a 20 μL reaction volume, containing1x SsoFast™ EvaGreen^®^ Supermix (Bio-Rad), equal concentration (100 nM) of each of the forward and reverse primers, and two μL of sample DNA. PCR was performed in a CFX96 Touch Real-Time PCR Detection System (Bio-Rad Laboratories) with an initial denaturation step at 95 °C for 4 min, followed by 40 cycles of 95 °C for 1 sec, 59 °C for 2 sec and 70 °C for 2 sec. The PCR product was then denatured at 95 °C (held for 30 sec), cooled to 65 °C (held for 60 sec), and melted from 65 °C to 85 °C with a 0.2 °C temperature increment every ten seconds with continuous data acquisition. The amplification plots and melting graphs were analysed using the CFX Manager^TM^ Software version 3.1 (Bio-Rad), and the corresponding curves displayed as negative first-derivative plots of fluorescence with respect to temperature. High-Resolution Melting (HRM) curve analysis was also used to analyze the data and melting profiles of the eight poxviruses using the Precision Melt Analysis^TM^ Software version 1.2 (Bio-Rad). Normalized melt curves and difference in curves were acquired by analyzing the active melt region separately for each virus genus by designating the corresponding pre-and post-melt regions.

### Limit of detection of the assay

Each plasmid was serially 10-fold diluted until 10^2^ viral copies were reached using Herring sperm DNA matrix (5 ng/mL) and kept at −20 °C until further analysis. Linearity of the assay was conducted for each viral species to determine the efficiency and dynamic range of the assay. Subsequently, the analytical sensitivity of the assay was assessed by amplifying six different concentrations of each plasmid, corresponding to each of the eight poxviruses (100, 80, 60, 40, 20 and 10 viral copies). Thus, the lower limit of detection (LOD) was determined by testing the diluted plasmids in pentaplicate, separately, at different days, on three separate occasions. The data from each PCR run were recorded and subjected to probit regression analysis using the STATGRAPHICS Centurion XV Version 15.2.12 Software package (StatPoint Technologies, Warrenton, VA, USA).

### Cross-platform compatibility test

The cross-platform compatibility of this assay was evaluated using various real-time PCR instruments. Thus, using the same PCR mix and protocol as in the BioRad CFX96 instrument, PCR and melting curve acquisition analysis was also conducted on the LightCycler^®^ 480 Real-Time PCR Systems (Roche), QuantStudio™ 6 Flex Real-Time PCR System (Life Technologies), and Rotor-Gene Q Real-Time PCR Cycler (Qiagen).

### Discriminating power of the test

The specificity and the discriminating power of the method was tested using DNA extracted from clinical samples of pox disease suspected animals and infected cell culture suspensions of isolates collected from sheep, goats, cattle, camels, cats and humans, originating from different geographical regions (Supplementary Table S1). Additionally, the analytical specificity of the method was evaluated by testing non-poxviral DNA of contagious caprine pleuropneumonia (CCPP), cDNA from peste des petits ruminants virus (PPRV) and foot and mouth disease virus (FMDV) (Supplementary Table S2). Samples were blinded to the operator and each sample was tested in duplicate and every PCR run included a no-template control and the appropriate positive controls (CPXV, CMLV, GTPV, SPPV, LSDV, ORFV, PCPV or BPSV), depending on the history of the test sample. The accuracy of the assay results was confirmed using previously established methods: species specific detection of CMLV targeting ATIP gene^7^,^33^, capripoxvirus genotyping methods^34^,^35^, species specific diagnostic PCR for ORFV^31^, and gene sequencing for CPXV^9^, PCPV^43^, and BPSV^44^. Based on a previous study, CCEV is most likely a subclade of PCPV which has adapted to camels as they are genetically closely related^17^. Our sequence analysis data showed all the isolates of CCEV in this study clustered closely with PCPV^45^, thus, we considered isolates of CCEV genotyped as PCPV.

One way ANOVA test was performed using STATGRAPHICS Centurion XV version 15.2.12 (Statpoint Technologies, Inc. Warrenton, Virginia) to determine whether the average Tm differences between the genotypes were significant. Additionally, box and whisker plots were constructed to illustrate the differences between the Tm of the eight different poxviruses.

## Additional Information

**How to cite this article:** Gelaye, E. *et al*. A novel HRM assay for the simultaneous detection and differentiation of eight poxviruses of medical and veterinary importance. *Sci. Rep.* **7**, 42892; doi: 10.1038/srep42892 (2017).

**Publisher's note:** Springer Nature remains neutral with regard to jurisdictional claims in published maps and institutional affiliations.

## Supplementary Material

Supplement Tables

## Figures and Tables

**Table 1:** Cross-platform analysis of the HRM assay.

Virus genotype	Real-Time PCR machines with Tm values			
	CFX96 (Bio-Rad)	LC480II (Roche)	QS6 (Life tech.)	RG-Q (Qiagen)
CPXV	72.20–72.40	72.70–72.75	72.75–72.88	73.44–73.47
CMLV	73.00–73.20	73.38–73.80	73.38–73.51	74.10–74.13
GTPV	75.60–75.80	76.49–76.53	76.38–76.48	77.14–77.20
SPPV	76.20–76.40	76.98–77.05	76.93–76.98	77.70–77.72
LSDV	77.20–77.40	78.00–78.03	77.84–77.96	78.70–78.73
ORFV	80.20–80.40	80.62–80.65	80.36–80.58	81.36–81.39
PCPV	81.20–81.40	82.08–82.10	81.82–81.95	82.70–82.71
BPSV	81.60–81.80	82.42–82.45	82.05–82.23	82.96–83.02

Different real-time PCR instruments used for assay evaluation with their respective amplicon melting temperature values indicated for the targeted eight poxvirus species.

**Table 2:** List of the oligonucleotides used in this study.

Primers’ ID	5′ → 3′sequence	PCR product length (bp)	Total G + C content (bp)
OPV-HRM-For	TAGGACTAGCCGCGGTAACTT	56	21
OPV-HRM-Rev	ACAAGATAGAAGCGATGGATACTT		
CaPV-HRM-For	TCCTGGCATTTTAAGTAATGGT	100	38
CaPV-HRM-Rev	GTCAGATATAAACCCGGCAAGTG		
PPV-HRM-For	TCGAAGATCTTGTCCAGGAAG	112	57
PPV-HRM-Rev	CCGAGAAGATCAACGAGGTC		

The names and sequences of the primers designed to amplify short fragments in the targeted virus genomes and the estimated PCR amplicon size and G + C content are presented.

**Table 3:** Poxviruses used for the production of positive control plasmids.

Genus	Genotype	Isolate name	Host	Origin
*Orthopoxvirus*	Cowpox virus	CPXV 72/93	Cattle	Austria
	Camelpox virus	CMLV Hadow/01/2012	Camel	Ethiopia
*Capripoxvirus*	Goatpox virus	GTPV Denizli	Goat	Turkey
	Sheeppox virus	SPPV Denizli	Sheep	Turkey
	Lumpy skin disease virus	LSDV Ismalia	Cattle	Egypt
*Parapoxvirus*	Orf virus	ORFV- DZ C-1	Sheep	Ethiopia
	Pseudocowpox virus	PCPV 2200/12	Human	Austria
	Bovine papular stomatitis virus	BPSV- Stamm M1	Cattle	Austria

The poxvirus genotype, isolates’ names, host and origins are presented.

**Table 4:** Primers used for production and verification of HRM control plasmids.

Primers’ ID	5′ → 3′sequence	PCR Product size (bp)
OPV-gp060f-For	CGGATGTAAAGACAATGAATGG	1207
OPV-gp060f-Rev	AAACGATTGACGTCCGAAAT	
CaPV-RPO147f-For	TCAATTAACTAGAATAAAGCAAGGAAA	1120
CaPV-RPO147f-Rev	TCCTCTCCCCTCTGGATCTT	
PaPV-VLTF3f-For	TCGGCAGCACGTACTCGAT	855
PaPV-VLTF3f-Rev	AGTGCTGGACCGCGAGAT	

The sequences of primers used for the amplification, cloning and sequencing of the full ssDNA-binding phosphoprotein gene (I3L) of orthopoxviruses, the partial RNA polymerase subunit gene (RPO147) of capripoxviruses, and the full late transcription factor gene (VLTF3) of parapoxviruses are presented with their estimated PCR product size are presented.

## References

[b1] Boyle K. & Traktman P. Viral Genome Replication. pp. 225–247 (Springer, 2009).

[b2] Maclachlan N. J. & Dubovi E. J. Fenner’s veterinary virology. Academic press (2010).

[b3] Dimmock N. J., Easton A. J. & Leppard K. N. The Process of Infection: IIA. The Replication of Viral DNA. Introduction to modern Virology 1–2 (2007).

[b4] Gubser C., Hue S., Kellam P. & Smith G. L. Poxvirus genomes: a phylogenetic analysis. J. Gen. Virol. 85, 105–117 (2004). 14718625 10.1099/vir.0.19565-0

[b5] Li Y., Meyer H., Zhao H. & Damon I. K. GC content-based pan-pox universal PCR assays for poxvirus detection. J. Clin. Microbiol. 48, 268–276 (2010). 19906902 10.1128/JCM.01697-09 PMC2812294

[b6] Bera B. C. . Zoonotic cases of camelpox infection in India. Vet Microbiol. 152, 29–38 (2011). 21571451 10.1016/j.vetmic.2011.04.010

[b7] OIE, U. N. Manual of Diagnostic Tests and Vaccines For Terrestrial Animals (Mammals, Birds and Bees). ed. (2012). 16642778

[b8] Hawranek T. . Feline orthopoxvirus infection transmitted from cat to human. J. Am. Acad. Dermatol. 49, 513–518 (2003). 12963921 10.1067/s0190-9622(03)00762-x

[b9] Carroll D. S. . Chasing Jenner’s vaccine: revisiting cowpox virus classification. PLoS. One. 6, e23086 (2011). 21858000 10.1371/journal.pone.0023086 PMC3152555

[b10] Dabrowski P. W., Radonic A., Kurth A. & Nitsche A. Genome-wide comparison of cowpox viruses reveals a new clade related to Variola virus. PLoS. One. 8, e79953 (2013). 24312452 10.1371/journal.pone.0079953 PMC3848979

[b11] Yan X. M. . An outbreak of sheep pox associated with goat poxvirus in Gansu province of China. Vet. Microbiol. 156, 425–428 (2012). 22169434 10.1016/j.vetmic.2011.11.015

[b12] Tuppurainen E. S. & Oura C. A. Review: lumpy skin disease: an emerging threat to Europe, the Middle East and Asia. Transbound. Emerg. Dis. 59, 40–48 (2012). 21749675 10.1111/j.1865-1682.2011.01242.x

[b13] Nougairede A. . Sheep-to-human transmission of Orf virus during Eid al-Adha religious practices, France. Emerg. Infect. Dis. 19, 102–105 (2013). 23260031 10.3201/eid1901.120421 PMC3557981

[b14] Zhang K., Liu Y., Kong H., Shang Y. & Liu X. Human infection with ORF virus from goats in China, 2012. Vector. Borne. Zoonotic. Dis. 14, 365-367 (2014). 24745915 10.1089/vbz.2013.1445 PMC4026105

[b15] Al-Salam S., Nowotny N., Sohail M. R., Kolodziejek J. & Berger T. G. Ecthyma contagiosum (orf)–report of a human case from the United Arab Emirates and review of the literature. J. Cutan. Pathol. 35, 603–607 (2008). 18201239 10.1111/j.1600-0560.2007.00857.x

[b16] Lederman E. . Zoonotic parapoxviruses detected in symptomatic cattle in Bangladesh. BMC. Res. Notes 7, 816 (2014). 25410770 10.1186/1756-0500-7-816 PMC4246640

[b17] Abubakr M. I. . Pseudocowpox virus: the etiological agent of contagious ecthyma (Auzdyk) in camels (Camelus dromedarius) in the Arabian peninsula. Vector. Borne. Zoonotic. Dis. 7, 257–260 (2007). 17627446 10.1089/vbz.2006.0627

[b18] Abrahao J. S. . Human Vaccinia virus and Pseudocowpox virus co-infection: clinical description and phylogenetic characterization. J. Clin. Virol. 48, 69–72 (2010). 20207192 10.1016/j.jcv.2010.02.001

[b19] Oguzoglu T. C., Koc B. T., Kirdeci A. & Tan M. T. Evidence of zoonotic pseudocowpox virus infection from a cattle in Turkey. Virusdisease. 25, 381–384 (2014). 25674608 10.1007/s13337-014-0214-z PMC4188210

[b20] Gori A., Cerboneschi M. & Tegli S. High-resolution melting analysis as a powerful tool to discriminate and genotype Pseudomonas savastanoi pathovars and strains. PLoS. One. 7, e30199 (2012). 22295075 10.1371/journal.pone.0030199 PMC3266268

[b21] Shchelkunov S. N., Shcherbakov D. N., Maksyutov R. A. & Gavrilova E. V. Species-specific identification of variola, monkeypox, cowpox, and vaccinia viruses by multiplex real-time PCR assay. J. Virol. Methods 175, 163–169 (2011). 21635922 10.1016/j.jviromet.2011.05.002 PMC9628778

[b22] Olson V. A. . Real-time PCR system for detection of orthopoxviruses and simultaneous identification of smallpox virus. J. Clin. Microbiol. 42, 1940–1946 (2004). 15131152 10.1128/JCM.42.5.1940-1946.2004 PMC404623

[b23] Gundry C. N. . Amplicon melting analysis with labeled primers: a closed-tube method for differentiating homozygotes and heterozygotes. Clin. Chem. 49, 396–406 (2003). 12600951 10.1373/49.3.396

[b24] Reed G. H. & Wittwer C. T. Sensitivity and specificity of single-nucleotide polymorphism scanning by high-resolution melting analysis. Clin. Chem. 50, 1748–1754 (2004). 15308590 10.1373/clinchem.2003.029751

[b25] Cho M. H., Ciulla D., Klanderman B. J., Raby B. A. & Silverman E. K. High-resolution melting curve analysis of genomic and whole-genome amplified DNA. Clin. Chem. 54, 2055–2058 (2008). 19042988 10.1373/clinchem.2008.109744 PMC2755063

[b26] Tajiri-Utagawa E., Hara M., Takahashi K., Watanabe M. & Wakita T. Development of a rapid high-throughput method for high-resolution melting analysis for routine detection and genotyping of noroviruses. J. Clin. Microbiol. 47, 435–440 (2009). 19073870 10.1128/JCM.01247-08 PMC2643702

[b27] Martino A., Mancuso T. & Rossi A. M. Application of high-resolution melting to large-scale, high-throughput SNP genotyping: a comparison with the TaqMan method. J. Biomol. Screen. 15, 623–629 (2010). 20371868 10.1177/1087057110365900

[b28] Ganopoulos I., Madesis P., Zambounis A. & Tsaftaris A. High-resolution melting analysis allowed fast and accurate closed-tube genotyping of Fusarium oxysporum formae speciales complex. FEMS Microbiol. Lett. 334, 16–21 (2012). 22670678 10.1111/j.1574-6968.2012.02610.x

[b29] Tong S. Y. & Giffard P. M. Microbiological applications of high-resolution melting analysis. J. Clin. Microbiol. 50, 3418–3421 (2012). 22875887 10.1128/JCM.01709-12 PMC3486245

[b30] Hata A., Kitajima M., Tajiri-Utagawa E. & Katayama H. Development of a high resolution melting analysis for detection and differentiation of human astroviruses. J. Virol. Methods 200, 29–34 (2014). 24509176 10.1016/j.jviromet.2014.01.023

[b31] Torfason E. G. & Gunadottir S. Polymerase chain reaction for laboratory diagnosis of orf virus infections. J. Clin. Virol. 24, 79–84 (2002). 11744431 10.1016/s1386-6532(01)00232-3

[b32] Gelaye E. . Molecular characterization of orf virus from sheep and goats in Ethiopia, 2008–2013. Virology journal 13, 1 (2016). 26923232 10.1186/s12985-016-0489-3 PMC4770539

[b33] Meyer H., Pfeffer M. & Rziha H. J. Sequence alterations within and downstream of the A-type inclusion protein genes allow differentiation of Orthopoxvirus species by polymerase chain reaction. J. Gen. Virol. 75 (Pt 8), 1975–1981 (1994). 8046400 10.1099/0022-1317-75-8-1975

[b34] Lamien C. E. . Real time PCR method for simultaneous detection, quantitation and differentiation of capripoxviruses. J. Virol. Methods 171, 134–140 (2011). 21029751 10.1016/j.jviromet.2010.10.014

[b35] Gelaye E. . Development of a cost-effective method for capripoxvirus genotyping using snapback primer and dsDNA intercalating dye. PLoS. One. 8, e75971 (2013). 24116084 10.1371/journal.pone.0075971 PMC3792100

[b36] Zhou T. . Phylogenetic analysis of Chinese sheeppox and goatpox virus isolates. Virol. J. 9, 25 (2012). 22264255 10.1186/1743-422X-9-25 PMC3398307

[b37] Tuppurainen E. S. . Characterization of sheep pox virus vaccine for cattle against lumpy skin disease virus. Antiviral Res. 109, 1–6 (2014). 24973760 10.1016/j.antiviral.2014.06.009 PMC4149609

[b38] Tuppurainen E. S. . Review: Capripoxvirus Diseases: Current Status and Opportunities for Control. Transbound. Emerg. Dis. (2015). 10.1111/tbed.12444 PMC5434826 26564428

[b39] Scagliarini A. . Orf in South Africa: endemic but neglected. Onderstepoort J. Vet. Res. 79, E1–E8 (2012). 10.4102/ojvr.v79i1.499 23327326

[b40] Zhao H., Wilkins K., Damon I. K. & Li Y. Specific qPCR assays for the detection of orf virus, pseudocowpox virus and bovine papular stomatitis virus. J. Virol. Methods 194, 229–234 (2013). 24035807 10.1016/j.jviromet.2013.08.027

[b41] Venkatesan G., Balamurugan V. & Bhanuprakash V. Multiplex PCR for simultaneous detection and differentiation of sheeppox, goatpox and orf viruses from clinical samples of sheep and goats. J. Virol. Methods 195, 1–8 (2014). 24134940 10.1016/j.jviromet.2013.10.009

[b42] Hall T. A. BioEdit: a user-friendly biological sequence alignment editor and analysis program for Windows 95/98/NT. Nucleic acids symposium series 41, 95–98. Ref Type: Conference Proceeding 1999).

[b43] Hautaniemi M. . The genome of pseudocowpoxvirus: comparison of a reindeer isolate and a reference strain. J. Gen. Virol. 91, 1560–1576 (2010). 20107016 10.1099/vir.0.018374-0

[b44] Delhon G. . Genomes of the parapoxviruses ORF virus and bovine papular stomatitis virus. J. Virol. 78, 168–177 (2004). 14671098 10.1128/JVI.78.1.168-177.2004 PMC303426

[b45] Gelaye E. . Genetic characterization of poxviruses in Camelus dromedarius in Ethiopia, 2011-2014. Antiviral Research 134, 17–25 (2016). 27544702 10.1016/j.antiviral.2016.08.016

