# Supplementary material for: A novel HRM assay for the simultaneous detection and differentiation of eight poxviruses of medical and veterinary importance
Source: Sci Rep. 2017 Feb 20;7:42892. doi: 10.1038/srep42892 (PMC5316968; doi:10.1038/srep42892)
Supplement: Supplement Tables [file srep42892-s1.pdf]

A novel HRM assay for the simultaneous detection and differentiation of eight poxviruses of medical and veterinary importance

Esayas Gelaye, Lukas Mach, Jolanta Kolodziejek, Reingard Grabherr, Angelika Loitsch, Jenna E. Achenbach, Norbert Nowotny, Adama Diallo and Charles Euloge Lamien

Supplementary information

Supplementary table S1. Poxvirus samples tested using the HRM assay. The name and origin of each isolate and the host, from which the tissue samples were taken, are indicated, together with the Tm and the genotype detected by HRM.

SI No	Strain name	Country/ Origin	Sample type	Host	Amplicon Tm	Genotype	Status
1	B624/2010	Kenya	Skin scraping	Cattle	77.40	LSDV	known
2	B291/2007	Kenya	Nodules	Cattle	77.40	LSDV	known
3	B3383/2011	Kenya	Nodules	Cattle	77.20	LSDV	known
4	O58/2011	Kenya	Skin scraping	Sheep	75.80	GTPV	known
5	G143/2009	Kenya	Skin scraping	Goat	75.60	GTPV	known
6	O59/2011	Kenya	Skin scraping	Sheep	75.60	GTPV	known
7	MOG/SP/T/1/2006	Mongolia	Skin scraping	Sheep	76.40	SPPV	known
8	MOG/SP/T/2/2006	Mongolia	Skin scraping	Sheep	76.20	SPPV	known
9	MOG/SP/T/3/2007	Mongolia	Skin scraping	Sheep	76.20	SPPV	known
10	MOG/GP/T/4/2008	Mongolia	Skin scraping	Goat	75.60	GTPV	known

11	MOG/GP/T/5/2008	Mongolia	Skin scraping	Goat	75.60	GTPV	known
12	MOG/GP/T/6/2008	Mongolia	Skin scraping	Goat	75.60	GTPV	known
13	Poumon 185M	Mali	Skin scraping	Sheep	76.20	SPPV	known
14	LSDV Massalamia	Sudan	Skin scraping	Cattle	77.20	LSDV	known
15	GTPV Denizli	Turkey	Skin scraping	Goat	75.80	GTPV	known
16	BPSV Stamm M1	Germany	Cell culture	Cattle	81.60	BPSV	known
17	CE 030ODV	Germany	Cell culture	Sheep	80.40	ORFV	known
18	ORFV D1701	Germany	Cell culture	Sheep	80.40	ORFV	known
19	GTPV Ghana	Ghana	Cell culture	Goat	75.60	GTPV	known
20	GTPV Bangladesh	Bangladesh	Cell culture	Goat	75.60	GTPV	known
21	KS-1	Kenya	Cell culture	Sheep	77.20	LSDV	known
22	SPPV/98 Darica	Turkey	Cell culture	Sheep	76.20	SPPV	known
23	SPPV HSL	Austria	Cell culture	Sheep	76.20	SPPV	known
24	SPPV Denizli	Turkey	Cell culture	Sheep	76.20	SPPV	known
25	SPPV Vaccine	Morocco	Cell culture	Vaccine	76.20	SPPV	known
26	GTPV Oman	Oman	Cell culture	Goat	75.60	GTPV	known
27	SPPV Illizi	Algeria	Cell culture	Sheep	76.00	SPPV	known
28	GTPV Gorgan	Iraq	Cell culture	Goat	75.60	GTPV	known
29	SPPV Djelfa	Algeria	Cell culture	Sheep	76.20	SPPV	known
30	SPPV Sivas	Turkey	Cell culture	Sheep	76.00	SPPV	known
31	GTPV Sudan	Sudan	Cell culture	Goat	76.20	SPPV	known
32	SPPV Corum	Turkey	Cell culture	Sheep	76.20	SPPV	known
33	GTPV Yemen	Yemen	Cell culture	Goat	75.80	GTPV	known
34	LSDV Sinnar	Sudan	Cell culture	Cattle	77.20	LSDV	known
35	LSDV North Sudan	Sudan	Cell culture	Cattle	77.20	LSDV	known
36	LSDV Ismalia	Egypt	Cell culture	Cattle	77.20	LSDV	known
37	OB3	Germany	Cell culture	Sheep	80.40	ORFV	known
38	LSDV Sundus	Sudan	Cell culture	Cattle	77.20	LSDV	known
39	SPPV Van	Turkey	Cell culture	Sheep	76.20	SPPV	known

40	SPPV Soba	Sudan	Cell culture	Sheep	76.20	SPPV	known
41	SPPV Oman	Oman	Cell culture	Sheep	75.60	GTPV	known
42	SPPV Algeria	Algeria	Cell culture	Sheep	76.20	SPPV	known
43	GTPV Saudi Arabia	Saudi Arabia	Cell culture	Goat	76.20	SPPV	known
44	ORFV 01/2014	Dondwe-Tanzania	Nasal swab	Goat	80.40	ORFV	Blind
45	ORFV 02/2014	Dondwe-Tanzania	Nasal swab	Goat	80.40	ORFV	Blind
46	Fafan/01/2012	Ethiopia	Skin scraping	Camel	73.00	CMLV	Blind
47	Fafan/02/2012	Ethiopia	Skin scraping	Camel	73.00	CMLV	Blind
48	Fafan/03/2012	Ethiopia	Skin scraping	Camel	81.40	PCPV	Blind
49	Golajo/01/2012	Ethiopia	Skin scraping	Camel	73.00	CMLV	Blind
50	Golajo/02/2012	Ethiopia	Skin scraping	Camel	72.80/81.40	CMLV/PCPV	Blind
51	Golajo/03/2012	Ethiopia	Skin scraping	Camel	73.00	CMLV	Blind
52	Hordha/01/2011	Ethiopia	Skin scraping	Camel	72.80/81.40	CMLV/PCPV	Blind
53	Hordha/02/2011	Ethiopia	Skin scraping	Camel	73.00	CMLV	Blind
54	Hadow/01/2012	Ethiopia	Skin scraping	Camel	73.00	CMLV	Blind
55	Hadow/02/2012	Ethiopia	Skin scraping	Camel	73.00	CMLV	Blind
56	Chifra H1/2011	Ethiopia	Skin scraping	Camel	81.40	PCPV	Blind
57	Chifra H2/2011	Ethiopia	Skin scraping	Camel	81.40	PCPV	Blind
58	Chifra H3/2011	Ethiopia	Skin scraping	Camel	81.40	PCPV	Blind
59	Chifra H4/2011	Ethiopia	Skin scraping	Camel	81.40	PCPV	Blind
60	Chifra H5/2011	Ethiopia	Skin scraping	Camel	81.40	PCPV	Blind
61	Chifra C-532/2011	Ethiopia	Skin scraping	Camel	81.40	PCPV	Blind
62	Borena VR352/14 C-1	Ethiopia	Skin scraping	Camel	73.00	CMLV	Blind
63	Borena VR352/14 C-2	Ethiopia	Skin scraping	Camel	72.80	CMLV	Blind
64	Borena VR352/14 C-3	Ethiopia	Skin scraping	Camel	73.00	CMLV	Blind
65	Borena VR352/14 C-4	Ethiopia	Skin scraping	Camel	73.00	CMLV	Blind
66	Borena VR352/14 C-5	Ethiopia	Skin scraping	Camel	73.00	CMLV	Blind
67	Borena VR352/14 C-6	Ethiopia	Skin scraping	Camel	72.80	CMLV	Blind
68	Borena VR352/14 C-A	Ethiopia	Skin scraping	Camel	73.00	CMLV	Blind

69	Borena VR352/14 C-B	Ethiopia	Skin scraping	Camel	72.80	CMLV	Blind
70	Borena VR352/14 C-C	Ethiopia	Skin scraping	Camel	73.00	CMLV	Blind
71	Borena VR352/14 C-D	Ethiopia	Skin scraping	Camel	72.80	CMLV	Blind
72	Borena VR352/14 C-BD1	Ethiopia	Skin scraping	Camel	73.00	CMLV	Blind
73	Borena VR352/14 C-BD2	Ethiopia	Skin scraping	Camel	72.80	CMLV	Blind
74	Borena VR352/14 C-BD3	Ethiopia	Skin scraping	Camel	73.00	CMLV	Blind
75	Borena VR352/14 C-BD4	Ethiopia	Skin scraping	Camel	73.00	CMLV	Blind
76	Borena C-HDG1 VR389/14	Ethiopia	Skin scraping	Camel	72.80	CMLV	Blind
77	Borena C-HDG2 VR389/14	Ethiopia	Skin scraping	Camel	72.80	CMLV	Blind
78	Borena C-HMA1 VR389/14	Ethiopia	Skin scraping	Camel	72.80	CMLV	Blind
79	Borena C-HMA2 VR389/14	Ethiopia	Skin scraping	Camel	72.80	CMLV	Blind
80	Borena C-2 VR387/14	Ethiopia	Skin scraping	Camel	81.20	PCPV	Blind
81	CaPV Adama 1	Ethiopia	Skin scraping	Sheep	75.80	GTPV	known
82	CaPV Adama 2	Ethiopia	Skin scraping	Sheep	75.40	GTPV	known
83	CaPV Adama 3	Ethiopia	Skin scraping	Sheep	75.60	GTPV	known
84	CaPV Adama 4	Ethiopia	Skin scraping	Sheep	75.60	GTPV	known
85	CaPV D/Berhan 1	Ethiopia	Skin scraping	Sheep	75.60	GTPV	known
86	CaPV D/Berhan 2	Ethiopia	Skin scraping	Sheep	75.60	GTPV	known
87	CaPV Mojo A	Ethiopia	Skin scraping	Sheep	75.60	GTPV	known
88	CaPV Mojo C	Ethiopia	Skin scraping	Sheep	75.80	GTPV	known
89	CaPV Fiche 1	Ethiopia	Skin scraping	Sheep	75.80	GTPV	known
90	CaPV Fiche 2	Ethiopia	Skin scraping	Sheep	75.80	GTPV	known
91	CaPV Assosa 1	Ethiopia	Skin scraping	Goat	75.60	GTPV	known
92	CaPV Assosa 2	Ethiopia	Skin scraping	Goat	75.80	GTPV	known
93	CaPV Andassa C-3	Ethiopia	Skin scraping	Cattle	77.40	LSDV	known
94	CaPV Andassa C-4	Ethiopia	Skin scraping	Cattle	77.40	LSDV	known
95	CaPV Andassa C-5	Ethiopia	Skin scraping	Cattle	77.40	LSDV	known
96	Borena VR386/14 C-1	Ethiopia	Skin scraping	Camel	81.20	PCPV	Blind
97	Borena VR386/14 C-2	Ethiopia	Skin scraping	Camel	81.20	PCPV	Blind

98	Borena VR386/14 C-3	Ethiopia	Skin scraping	Camel	81.20	PCPV	Blind
99	Borena VR386/14 C-4	Ethiopia	Skin scraping	Camel	81.20	PCPV	Blind
100	Borena VR386/14 C-5	Ethiopia	Skin scraping	Camel	81.20	PCPV	Blind
101	Borena VR386/14 C-6	Ethiopia	Skin scraping	Camel	81.20	PCPV	Blind
102	Borena VR386/14 C-7	Ethiopia	Skin scraping	Camel	81.20	PCPV	Blind
103	Borena VR386/14 C-8	Ethiopia	Skin scraping	Camel	81.20	PCPV	Blind
104	Borena VR386/14 C-9	Ethiopia	Skin scraping	Camel	81.20	PCPV	Blind
105	Borena VR386/14 C-10	Ethiopia	Skin scraping	Camel	81.20	PCPV	Blind
106	LSD NS H/Abote 04	Ethiopia	Nasal swab	Cattle	77.20	LSDV	Blind
107	LSD NS H/Abote 05	Ethiopia	Nasal swab	Cattle	77.20	LSDV	Blind
108	LSD NS H/Abote 06	Ethiopia	Nasal swab	Cattle	77.20	LSDV	Blind
109	LSD NS H/Abote 07	Ethiopia	Nasal swab	Cattle	77.20	LSDV	Blind
110	LSD NS H/Abote 08	Ethiopia	Nasal swab	Cattle	77.20	LSDV	Blind
111	LSD NS Kuyu 18	Ethiopia	Nasal swab	Cattle	77.20	LSDV	Blind
112	LSD NS Kuyu 19	Ethiopia	Nasal swab	Cattle	77.20	LSDV	Blind
113	LSD NS Kuyu 20	Ethiopia	Nasal swab	Cattle	77.20	LSDV	Blind
114	LSD NS Kuyu 21	Ethiopia	Nasal swab	Cattle	77.20	LSDV	Blind
115	LSD NS Kuyu 22	Ethiopia	Nasal swab	Cattle	77.20	LSDV	Blind
116	LSD NS Kuyu 23	Ethiopia	Nasal swab	Cattle	77.20	LSDV	Blind
117	LSD NS Kuyu 24	Ethiopia	Nasal swab	Cattle	77.20	LSDV	Blind
118	LSD NS Kuyu 25	Ethiopia	Nasal swab	Cattle	77.20	LSDV	Blind
119	LSD NS Kuyu 27	Ethiopia	Nasal swab	Cattle	77.20	LSDV	Blind
120	LSD LN Kuyu 18	Ethiopia	Lymph Node Aspirate	Cattle	77.20	LSDV	Blind
121	LSD LN Kuyu 19	Ethiopia	Lymph Node Aspirate	Cattle	77.20	LSDV	Blind
122	LSD LN Kuyu 23	Ethiopia	Lymph Node Aspirate	Cattle	77.20	LSDV	Blind
123	LSD NS W/Jarso 09	Ethiopia	Nasal swab	Cattle	77.20	LSDV	Blind
124	LSD NS W/Jarso 11	Ethiopia	Nasal swab	Cattle	77.20	LSDV	Blind
125	LSD NS W/Jarso 33	Ethiopia	Nasal swab	Cattle	77.20	LSDV	Blind
126	LSD NS W/Jarso 34	Ethiopia	Nasal swab	Cattle	77.20	LSDV	Blind

127	LSD NS W/Jarso 39	Ethiopia	Nasal swab	Cattle	77.20	LSDV	Blind
128	LSD NS W/Jarso 40	Ethiopia	Nasal swab	Cattle	77.20	LSDV	Blind
129	ORFV MB38/13 C-1	Ethiopia	Skin scraping	Sheep	80.40	ORFV	known
130	ORFV MB38/13 C-2	Ethiopia	Skin scraping	Sheep	80.40	ORFV	known
131	ORFV MB38/13 C-3	Ethiopia	Skin scraping	Sheep	80.40	ORFV	known
132	ORFV MB38/13 C-4	Ethiopia	Skin scraping	Sheep	80.40	ORFV	known
133	ORFV MB38/13 C-5	Ethiopia	Skin scraping	Goat	80.40	ORFV	known
134	ORFV MB38/13 C-6	Ethiopia	Skin scraping	Goat	80.40	ORFV	known
135	ORFV MB38/13 C-7	Ethiopia	Skin scraping	Goat	80.40	ORFV	known
136	ORFV MB38/13 C-8	Ethiopia	Skin scraping	Goat	80.40	ORFV	known
137	ORFV Adet C-1	Ethiopia	Skin scraping	Sheep	80.60	ORFV	known
138	ORFV Adet C-2	Ethiopia	Skin scraping	Sheep	80.60	ORFV	known
139	ORFV DZ C-1	Ethiopia	Skin scraping	Sheep	80.40	ORFV	known
140	ORFV DZ C-2	Ethiopia	Skin scraping	Sheep	80.40	ORFV	known
141	ORFV ATARC 2008 C-1	Ethiopia	Skin scraping	Sheep	80.40	ORFV	known
142	ORFV ATARC 2008 C-2	Ethiopia	Skin scraping	Sheep	80.40	ORFV	known
143	ORFV ATARC 2010 C-1	Ethiopia	Skin scraping	Sheep	80.40	ORFV	known
144	ORFV ATARC 2010 C-2	Ethiopia	Skin scraping	Sheep	80.40	ORFV	known
145	CaPV Ambo N swab C-3	Ethiopia	Nasal swab	Cattle	77.20	LSDV	Blind
146	CaPV Ambo N swab C-4	Ethiopia	Nasal swab	Cattle	77.20	LSDV	Blind
147	CaPV Ambo N swab C-5	Ethiopia	Nasal swab	Cattle	77.20	LSDV	Blind
148	CaPV Ambo T susp C-2	Ethiopia	Skin scraping	Cattle	77.20	LSDV	Blind
149	CaPV Ambo T susp C-7758	Ethiopia	Skin scraping	Cattle	77.20	LSDV	Blind
150	CaPV Ambo T susp C-7815	Ethiopia	Skin scraping	Cattle	77.20	LSDV	Blind
151	CaPV Ambo LN B-506	Ethiopia	Lymph Node Aspirate	Cattle	77.20	LSDV	Blind
152	CaPV Ambo LN 061	Ethiopia	Lymph Node Aspirate	Cattle	77.00	LSDV	Blind
153	CaPV Ambo LN 057	Ethiopia	Lymph Node Aspirate	Cattle	77.00	LSDV	Blind
154	CaPV Ambo LN B-406	Ethiopia	Lymph Node Aspirate	Cattle	77.00	LSDV	Blind
155	CaPV Tigray C-1	Ethiopia	Skin scraping	Goat	75.60	GTPV	Blind

156	CaPV Tigray C-2	Ethiopia	Skin scraping	Goat	75.60	GTPV	Blind
157	CaPV Asella WJ 12	Ethiopia	Nasal swab	Cattle	77.00	LSDV	Blind
158	CaPV Asella WJ 13	Ethiopia	Nasal swab	Cattle	77.00	LSDV	Blind
159	CaPV Asella WJ 14	Ethiopia	Nasal swab	Cattle	77.00	LSDV	Blind
160	CaPV Asella WJ 15	Ethiopia	Nasal swab	Cattle	77.00	LSDV	Blind
161	CaPV Asella WJ 16	Ethiopia	Nasal swab	Cattle	77.00	LSDV	Blind
162	CaPV Asella WJ 11	Ethiopia	Lymph Node Aspirate	Cattle	77.00	LSDV	Blind
163	CaPV Asella WJ 13	Ethiopia	Lymph Node Aspirate	Cattle	77.00	LSDV	Blind
164	CaPV Asella WJ 14	Ethiopia	Lymph Node Aspirate	Cattle	77.00	LSDV	Blind
165	CaPV Asella WJ 15	Ethiopia	Lymph Node Aspirate	Cattle	77.20	LSDV	Blind
166	CaPV Asella WJ 16	Ethiopia	Lymph Node Aspirate	Cattle	77.00	LSDV	Blind
167	CaPV B/Dar Lab C-1	Ethiopia	Nasal swab	Cattle	77.00	LSDV	Blind
168	CaPV B/Dar Lab C-3	Ethiopia	Nasal swab	Cattle	77.20	LSDV	Blind
169	CaPV B/Dar Lab C-6	Ethiopia	Occular swab	Cattle	77.00	LSDV	Blind
170	CaPV B/Dar Lab C-1	Ethiopia	Lymph Node Aspirate	Cattle	77.00	LSDV	Blind
171	CaPV B/Dar Lab C-3	Ethiopia	Lymph Node Aspirate	Cattle	77.00	LSDV	Blind
172	CaPV Tigray C-034	Ethiopia	Skin scraping	Cattle	77.00	LSDV	Blind
173	CaPV Tigray C-035	Ethiopia	Skin scraping	Cattle	77.00	LSDV	Blind
174	CaPV Ambo 501	Ethiopia	Nasal swab	Cattle	77.20	LSDV	Blind
175	CaPV Ambo B-047	Ethiopia	Nasal swab	Cattle	77.00	LSDV	Blind
176	CaPV Ambo B-405	Ethiopia	Nasal swab	Cattle	77.00	LSDV	Blind
177	CaPV Ambo B-508	Ethiopia	Nasal swab	Cattle	77.00	LSDV	Blind
178	CaPV Ambo B-406	Ethiopia	Nasal swab	Cattle	77.20	LSDV	Blind
179	CaPV Ambo C-6	Ethiopia	Nasal swab	Cattle	77.00	LSDV	Blind
180	CaPV Ambo B-06	Ethiopia	Nasal swab	Cattle	77.00	LSDV	Blind
181	CaPV Ambo B-40	Ethiopia	Nasal swab	Cattle	77.00	LSDV	Blind
182	CaPV Ambo B-407	Ethiopia	Nasal swab	Cattle	77.00	LSDV	Blind
183	CaPV Akaki	Ethiopia	Skin scraping	Sheep	75.80	GTPV	known
184	NVI CaPV vaccine	Ethiopia	Cell culture	Vaccine	77.40	LSDV	known

185	CaPV Benshangul	Ethiopia	Skin scraping	Goat	75.80	GTPV	known
186	CaPV Awi C-1	Ethiopia	Skin scraping	Sheep	75.80	GTPV	known
187	CaPV Awi C-2	Ethiopia	Skin scraping	Sheep	75.80	GTPV	known
188	CaPV Awi C-3	Ethiopia	Skin scraping	Sheep	75.80	GTPV	known
189	CaPV Awi C-4	Ethiopia	Skin scraping	Sheep	75.80	GTPV	known
190	CaPV Awi C-5	Ethiopia	Skin scraping	Sheep	75.80	GTPV	known
191	CaPV Awi C-6	Ethiopia	Skin scraping	Sheep	75.80	GTPV	known
192	CaPV FairField	Ethiopia	Skin scraping	Cattle	77.40	LSDV	known
193	CaPV Kajima	Ethiopia	Skin scraping	Cattle	77.40	LSDV	known
194	CaPV EIAR	Ethiopia	Skin scraping	Cattle	77.40	LSDV	known
195	CaPV EDLTI	Ethiopia	Skin scraping	Cattle	77.20	LSDV	known
196	CaPV Metekel	Ethiopia	Skin scraping	Sheep	75.80	GTPV	known
197	CaPV NVI	Ethiopia	Skin scraping	Goat	75.80	GTPV	known
198	CaPV Adama C-1	Ethiopia	Skin scraping	Cattle	77.40	LSDV	known
199	CaPV Adama C-2	Ethiopia	Skin scraping	Cattle	77.40	LSDV	known
200	CaPV Mojo C-1	Ethiopia	Skin scraping	Cattle	77.40	LSDV	known
201	CaPV Mojo C-2	Ethiopia	Skin scraping	Cattle	77.40	LSDV	known
202	CaPV Wenji C-1	Ethiopia	Skin scraping	Cattle	77.40	LSDV	known
203	CaPV Wenji C-2	Ethiopia	Skin scraping	Cattle	77.40	LSDV	known
204	CaPV Wenji C-3	Ethiopia	Skin scraping	Cattle	77.40	LSDV	known
205	CaPV Andassa C-1	Ethiopia	Skin scraping	Cattle	77.40	LSDV	known
206	CaPV Andassa C-2	Ethiopia	Skin scraping	Cattle	77.40	LSDV	known
207	CaPV Andassa C-3	Ethiopia	Skin scraping	Cattle	77.40	LSDV	known
208	443/02	Austria	Skin scraping	Feline	71.80	CPXV	known
209	548/07	Austria	Skin scraping	Cattle	72.00	CPXV	known
210	531/92	Austria	Skin scraping	Feline	72.20	CPXV	known
211	2200/12	Austria	Skin scraping	Human	81.20	PCPV	known
212	1967/09	Austria	Skin scraping	Human	80.40	ORFV	known
213	72/93	ATCC VR 302	Cell culture	Cattle	72.20	CPXV	known

214	25/07	Austria	Skin scraping	Human	80.40	ORFV	known
215	70/10	Austria	Skin scraping	Human	72.00	CPXV	known
216	73/93	ATCC VR 156	Cell culture	Human	72.20	CPXV	known
217	563/10	Austria	Skin scraping	Feline	71.80	CPXV	known
218	PPV 603625	UK (IAH)	Skin scraping	Cattle	81.20	PCPV	known
219	CaPV 2008 C-2	Senegal	Skin scraping	Cattle	77.40	LSDV	known
220	CaPV 2008 C-3	Senegal	Skin scraping	Cattle	77.40	LSDV	known
221	CaPV 2008 C-4	Senegal	Skin scraping	Cattle	77.20	LSDV	known
222	CaPV 2008 C-6	Senegal	Skin scraping	Cattle	77.40	LSDV	known
223	CaPV 2011 C-10	Senegal	Skin scraping	Cattle	77.40	LSDV	known
224	RM 65-Romania	PANVAC-AU	Cell culture	Vaccine	76.00	SPPV	known
225	SPPV Vaccine-Egypt	PANVAC-AU	Cell culture	Vaccine	76.40	SPPV	known
226	KS-1-Kenya	PANVAC-AU	Cell culture	Vaccine	77.40	LSDV	known
227	PANVAC C-2	PANVAC-AU	Cell culture	Vaccine	77.40	LSDV	known
228	PANVAC C-3	PANVAC-AU	Cell culture	Vaccine	77.40	LSDV	known
229	PANVAC C-4	PANVAC-AU	Cell culture	Vaccine	77.40	LSDV	known
230	PANVAC C-5	PANVAC-AU	Cell culture	Vaccine	77.40	LSDV	known
231	PANVAC C-6	PANVAC-AU	Cell culture	Vaccine	76.40	SPPV	known
232	PANVAC C-7	PANVAC-AU	Cell culture	Vaccine	77.40	LSDV	known
233	PANVAC C-8	PANVAC-AU	Cell culture	Vaccine	77.40	LSDV	known
234	PANVAC C-9	PANVAC-AU	Cell culture	Vaccine	77.40	LSDV	known
235	PANVAC C-10	PANVAC-AU	Cell culture	Vaccine	77.40	LSDV	known
236	CaPV C-2	Uganda	Swab	Bovine	77.20	LSDV	Blind
237	CaPV C-3	Uganda	Swab	Bovine	77.20	LSDV	Blind
238	CaPV C-4	Uganda	Swab	Bovine	77.20	LSDV	Blind
239	CaPV C-6	Uganda	Swab	Bovine	77.40	LSDV	Blind
240	CaPV C-7	Uganda	Swab	Bovine	77.40	LSDV	Blind
241	CaPV C-8	Uganda	Swab	Bovine	77.20	LSDV	Blind
242	CaPV C-9	Uganda	Skin scraping	Bovine	77.40	LSDV	Blind

243	CaPV C-10	Uganda	Skin scraping	Bovine	77.40	LSDV	Blind
244	CaPV C-11	Uganda	Skin scraping	Bovine	77.20	LSDV	Blind
245	CaPV C-1	Ethiopia	Skin scraping	Cattle	77.40	LSDV	Blind
246	CaPV C-2	Ethiopia	Skin scraping	Cattle	77.20	LSDV	Blind
247	CaPV C-3	Ethiopia	Skin scraping	Cattle	77.20	LSDV	Blind
248	CaPV C-4	Ethiopia	Skin scraping	Cattle	77.40	LSDV	Blind
249	CaPV C-6	Ethiopia	Skin scraping	Cattle	77.40	LSDV	Blind
250	CaPV C-7	Ethiopia	Skin scraping	Cattle	77.20	LSDV	Blind
251	CaPV C-8	Ethiopia	Skin scraping	Cattle	77.20	LSDV	Blind
252	CaPV C-9	Ethiopia	Skin scraping	Cattle	77.40	LSDV	Blind
253	CaPV C-10	Ethiopia	Skin scraping	Cattle	77.40	LSDV	Blind
254	CaPV C-11	Ethiopia	Skin scraping	Cattle	77.40	LSDV	Blind
255	CaPV C-12	Ethiopia	Skin scraping	Cattle	77.20	LSDV	Blind
256	CaPV C-13	Ethiopia	Skin scraping	Goat	75.80	GTPV	Blind
257	CaPV C-14	Ethiopia	Skin scraping	Goat	75.80	GTPV	Blind
258	CaPV C-15	Ethiopia	Skin scraping	Goat	75.80	GTPV	Blind
259	CaPV C-17	Ethiopia	Skin scraping	Goat	75.60	GTPV	Blind
260	CaPV C-18	Ethiopia	Skin scraping	Goat	75.80	GTPV	Blind
261	ORF Msongola	Tanzania	Scab scraping	Goat	80.40	ORFV	Blind
262	ORF Kakubiro	Tanzania	Scab scraping	Goat	80.40	ORFV	Blind
263	ORF Dondwe1	Tanzania	Scab scraping	Goat	80.40	ORFV	Blind
264	ORF Dondwe2	Tanzania	Scab scraping	Goat	80.40	ORFV	Blind
265	ORF Mwanzomgumu	Tanzania	Scab scraping	Goat	80.40	ORFV	Blind
266	ORF Kimanga1	Tanzania	Scab scraping	Goat	80.40	ORFV	Blind
267	ORF Kimanga2	Tanzania	Scab scraping	Goat	80.40	ORFV	Blind
268	ORF Mapinga1	Tanzania	Scab scraping	Goat	80.40	ORFV	Blind
269	ORF Mapinga2	Tanzania	Scab scraping	Goat	80.40	ORFV	Blind
270	ORF Kyela2013	Tanzania	Scab scraping	Goat	80.40	ORFV	Blind
271	BPSV	Austria	Cell culture	Cattle	81.60	BPSV	known

Supplement table S2. Non-pox virus sample using the HRM assay.

SI No.	Strain name	Country of origin	Sample type	Host	Tm	Genotype
1	PPRV Nigeria 75/1	Ethiopia	Vaccine	N/A	No amplification	None
2	cDNA PPRV	Ethiopia	Pathological lesions	Goat	No amplification	None
3	cDNA PPRV	Ethiopia	Pathological lesions	Goat	No amplification	None
4	cDNA PPRV	Ethiopia	Pathological lesions	Goat	No amplification	None
5	cDNA FMDV	Ethiopia	Vaccine	N/A	No amplification	None
6	cDNA FMDV	Ethiopia	Lesion scraping	Cattle	No amplification	None
7	cDNA FMDV	Ethiopia	Lesion scraping	Cattle	No amplification	None
8	cDNA FMDV	Ethiopia	Lesion scraping	Cattle	No amplification	None
9	cDNA FMDV	Ethiopia	Lesion scraping	Cattle	No amplification	None
10	DNA CCPP	Ethiopia	Vaccine	N/A	No amplification	None
11	DNA CCPP	Ethiopia	Pathological lesions	Goat	No amplification	None
12	DNA CCPP	Ethiopia	Pathological lesions	Goat	No amplification	None
13	DNA CCPP	Ethiopia	Pathological lesions	Goat	No amplification	None
14	DNA CCPP	Ethiopia	Pathological lesions	Goat	No amplification	None
